# Wheat Straw Return Influences Nitrogen-Cycling and Pathogen Associated Soil Microbiota in a Wheat–Soybean Rotation System

**DOI:** 10.3389/fmicb.2019.01811

**Published:** 2019-08-08

**Authors:** Hongjun Yang, Jiaxin Ma, Zhenyang Rong, Dandan Zeng, Yuanchao Wang, Shuijin Hu, Wenwu Ye, Xiaobo Zheng

**Affiliations:** ^1^Department of Plant Pathology, Nanjing Agricultural University, Nanjing, China; ^2^The Key Laboratory of Integrated Management of Crop Diseases and Pests, Ministry of Education, Nanjing, China; ^3^College of Resources and Environmental Sciences, Nanjing Agricultural University, Nanjing, China; ^4^Department of Entomology and Plant Pathology, North Carolina State University, Raleigh, NC, United States

**Keywords:** wheat straw return, wheat–soybean rotation, soil bacterial and fungal community, 16S rRNA and ITS amplicon sequencing, nitrogen-cycling microbe, plant pathogen

## Abstract

Returning straw to soil is an effective way to sustain or improve soil quality and crop yields. However, a robust understanding of the impact of straw return on the composition of the soil microbial communities under field conditions has remained elusive. In this study, we characterized the effects of wheat straw return on soil bacterial and fungal communities in a wheat–soybean rotation system over a 3-year period, using Illumina-based 16S rRNA, and internal transcribed region (ITS) amplicon sequencing. Wheat straw return significantly affected the α-diversity of the soil bacterial, but not fungal, community. It enhanced the relative abundance of the bacterial phylum Proteobacteria and the fungal phylum Zygomycota, but reduced that of the bacterial phylum Acidobacteria, and the fungal phylum Ascomycota. Notably, it enriched the relative abundance of nitrogen-cycling bacterial genera such as *Bradyrhizobium* and *Rhizobium*. Preliminary analysis of soil chemical properties indicated that straw return soils had significantly higher total nitrogen (TN) contents than no straw return soils. In addition, the relative abundance of fungal genera containing pathogens was significantly lower in straw return soils relative to control soils, such as *Fusarium*, *Alternaria*, and *Myrothecium*. These results suggested a selection effect from the 3-year continuous straw return treatment and the soil bacterial and fungal communities were moderately changed.

## Introduction

Burning of wheat straw after harvest is common in rural areas, which is a major seasonal source of air pollution and haze (Marschner et al., 2011; Qu et al., 2012; Xing et al., 2018) and contributes to the content of particulate matter of ≤2.5 μm diameter (PM2.5) in China (Qu et al., 2012; Xing et al., 2018). China produces ca. 0.9 billion tons of crop straw per year, which is rich in organic matter and nutrients (Valdez-Vazquez et al., 2016; Lu et al., 2018; Ma et al., 2018). Thus, straw burning also causes great loss of renewable resources (Yang et al., 2016; Lu et al., 2018). Consequently, straw return has been promoted as a means of improving soil fertility and crop yield (Marschner et al., 2011; Guo et al., 2016; Chen Y. et al., 2017; Li et al., 2018), while mitigating particulate air pollution.

The environmental impact on soils of straw return have been well studied, including soil water potential (Yang et al., 2016), temperature (Yang et al., 2016), enzyme activities (Ji et al., 2014; Zhao et al., 2016), soil organic matter fractions (Zhao et al., 2016; Chen Z. et al., 2017; Karlsson et al., 2017), soil quality and crop productivity (Hansen et al., 2017), soil greenhouse gas emissions (Bao et al., 2016; Zhou et al., 2017), soil chemical properties (Yu et al., 2018), and soil microbial communities (Marschner et al., 2011; Chen Y. et al., 2017; Li et al., 2017; Maarastawi et al., 2018). These results provide basic understanding in terms of how straw return may change soil carbon retention, soil quality, and soil ecosystem functions, and revealed a number of positive consequences, such as reducing soil water potential, increasing soil temperature and the activities of hydrolytic enzymes, and enhancing soil microbial functional diversity. However, there were limited studies on the potential effect of straw return to soil-borne pathogens. Straw return was usually deemed to the main causal agent for the soil-borne diseases, by providing good circumstances for pathogen growth, propagation, and accumulation, which then resulted in disease epidemic (Zhen et al., 2009; Li et al., 2012; Qiao et al., 2013). Whereas, some studies indicated that straw return could improve soil ecological environment (Lenka and Lal, 2013; Peng et al., 2013; Yuan et al., 2014), increase amounts of antagonistic microbes, disturb pathogen growth, increase plant resistance to pathogens, and then control soil-borne plant diseases (Osunlaja, 1990; Bailey and Lazarovits, 2003; Zhen et al., 2009).

Due to a new environmental regulation that prohibits the burning of straw, wheat straw return has increasingly been adopted in the Huang-Huai region of China. Huang-Huai region covers a huge area in Shandong, Anhui, Jiangsu, and Henan provinces between the Yellow River and the Haihe River, and is one of the main soybean-producing areas in China and under a rotation of summer soybean and winter wheat. In this study, we used Illumina-based 16S rRNA and internal transcribed region (ITS) amplicon sequencing to characterize the effects of straw return on soil bacterial and fungal communities, respectively, based on a three-season field experiment conducted from 2015 to 2017 in a wheat–soybean cropping system in three sites of the Huang-Huai region. We addressed the following questions: What are the effects of short-term wheat straw return on the soil bacterial and fungal communities under this system? How do microbes involved in the soil nitrogen cycle and wheat/soybean diseases respond to straw return?

## Materials and Methods

### Field Trial and Sampling

A three-season field experiment was conducted from 2015 to 2017 in a wheat–soybean cropping system in three sites of Huang-Huai region of China, including those at Jining, Shandong Province (35°27′N, 116°35′E, sandy loam soil); Xuzhou, Jiangsu Province (34°17′N, 117°17′E, yellow loam sand soil); and Suzhou, Anhui Province (33°38′N, 117°05′E, mortar black soil) (Supplementary Figure S1A). At each location, we sampled two production fields with different treatments: no wheat straw return (N), i.e., artificial harvest of wheat, wheat stubble less than 5 cm in height, and all wheat straw removed from the field; and wheat straw return (R), i.e., all wheat straw was crushed into pieces and mulched in the soil by no tillage after wheat harvest every year. The biomass of wheat straw was about 5000 kg.hm^–2^. Each treatment area was 30 × 8 m and encompassed five replicate subplots (Supplementary Figure S1B). The wheat–soybean cropping system dominates all three sites, with winter wheat seeded in early October and harvested in early June, and summer soybean seeded in mid-June and harvested in late September. The winter wheat cultivar is Jimai 22 (super-high yield cultivar) and the summer soybean cultivar is Zhonghuang13 (high-protein cultivar); both are popular in Huang-Huai region of China. The plots received basal fertilization of 50 kg N-P_2_O_5_-K_2_O ha^–1^ and 15 kg urea ha^–1^ at wheat seeding, and 10 kg N-P_2_O_5_-K_2_O ha^–1^ were applied at soybean seeding. The agronomic management and fertilization regimes at the three sites were similar.

Bulk soil samples were collected twice annually in June and August from 2015 to 2017: at 0 day (pre-planting) and 60 days after planting. For each site, 10 subplots (480 m^2^; i.e., 5 replicates per treatment) were chosen and 9 cores (20 cm depth) were collected from each subplot in an S-formation sampling method using shovels (3.8 cm diameter). The 9 cores from each subplot were mixed to form one composite sample, resulting in 20 samples per site per year. In total, 180 soil samples were analyzed (3 locations × 2 treatments × 5 subplots per treatment × 3 years × 2 time points per year). The soil samples were placed into separate sterile plastic bags and transported to the laboratory on ice. Each soil sample was sieved through a 2 mm mesh to remove roots and plant detritus. The samples were stored at –20°C until required.

### Soil DNA Extraction

To minimize DNA extraction bias, DNA was extracted in quadruplicate from the composite soil samples. DNA was extracted from ca. 0.25 g bulk soil using the MoBio PowerSoil DNA Isolation Kit following the manufacturer’s protocol (MoBio Laboratories Inc., Carlsbad, CA, United States). The four DNA samples were pooled, and the DNA concentration and purity were quantified using a NanoDrop 1000 Spectrophotometer (Thermo Fisher Scientific).

### PCR Amplification, Library Preparation, and Sequencing

Targeted metagenomic profiling of the samples was performed as described previously (Caporaso et al., 2011; McGuire et al., 2013). The V4 region of the bacterial 16S rRNA gene was amplified using the primers 515F (5′-GTGCCAGCMGCCGCGGTAA-3′) and 806R (5′-GGACTACHVGGGTWTCTAAT-3′) (Peiffer et al., 2013; Edwards et al., 2015), and the ITS1 region of the fungal ITS was amplified using the barcode primers ITS1F (5′-CTTGGTCATTTAGAGGAAGTAA-3′) and ITS2 (5′-GCTGCGTTCTTCATCGATGC-3′) (Xiong et al., 2017; Yang et al., 2018). PCR reaction mixtures (25 μL) contained Enzymatics Veraseq 2.0 Master Mix (NEB Phusion High-Fidelity PCR Master Mix), 13 μL PCR-grade water, 10 μL Master Mix, 0.5 μL forward primer (10 μM), 0.5 μL reverse primer (10 μM), and 1 μL (30 ng) sample DNA. The reaction mixtures were placed in a PCR Thermal Cycler Dice (TaKaRa, Japan) and thermal cycle was performed as follows: 94°C for 1 min, followed by 30 cycles of 45 s at 94°C, 30 s at 56°C for 16S or 52°C for ITS, and 90 s at 72°C. A final extension at 72°C for 10 min was followed by a hold at 4°C. The PCR products were run on a 1% agarose gel to verify successful amplification. Unsuccessful reactions were repeated but removed from the experiment if unsuccessful a second time. PCR products were cleaned using the Agencourt AMPure XP magnetic beads purification system (Beckman Coulter) (Stark et al., 2015). The final library was quantified by determining the average molecule length using the Agilent 2100 Bioanalyzer (Agilent DNA 1000 Reagents) and by real-time quantitative PCR (qPCR) (EvaGreen^TM^). Qualified libraries were paired-end sequenced using the MiSeq System with the PE250 strategy (PE251 + 8 + 8 + 251; MiSeq Reagent Kit).

### Bioinformatics Analyses

Paired-end reads were generated on the Illumina MiSeq platform. The sequencing data were deposited in NCBI database (BioProject ID: PRJNA524011). After removing the adaptors and primer sequences, the raw sequences were assembled according to the unique barcode using QIIME (Caporaso et al., 2010). Split sequences for each sample were merged using FLASH (v. 1.2.11) (Magoc and Salzberg, 2011). The tags were clustered into operational taxonomic units (OTUs) with a 97% threshold using UPARSE (Edgar, 2013), and chimeras were filtered out using UCHIME (v. 4.2.40) (Edgar et al., 2011). Representative bacterial sequences were taxonomically classified using Ribosomal Database Project Classifier v. 2.2 against the Silva database (v. 128) (Quast et al., 2013), and representative fungal OTUs were classified using the UNITE database (v. 7.0) (Koljalg et al., 2013), with a cutoff confidence value of 0.6. OTUs not assigned to target species were removed from the data set.

Fungal and bacterial α-diversities were estimated by calculating the OTU richness and Shannon diversity indices in Mothur (v. 1.31.2)^1^. Principal coordinates analyses (PCoAs) on Bray–Curtis dissimilarities were performed using the “pcoa” function in the R package^2^. To test for significant differences between groups of samples, an analysis of similarity (ANOSIM) was performed in Vegan with 999 permutations based on Bray–Curtis distances between samples. For Mantel tests, the Bray-Curtis and Euclidean distances were used to construct dissimilarity matrices of communities and soil characteristics, respectively, using QIIME^3^. Mantel tests were used to calculate the correlations between the soil bacterial/fungal community composition and soil characteristics. The linear discriminant analysis (LDA) effect size (LEfSe) method was used to evaluate bacterial and fungal taxa significantly associated with wheat straw return (Segata et al., 2011). The LEfSe analyses were performed using the Galaxy web application and workflow framework^4^. The α-value threshold employed for the non-parametric factorial Kruskal–Wallis sum-rank test was 0.05, and the logarithmic LDA score threshold for feature discrimination was 2.0.

### Analyses of Soil Chemical Properties

As a preliminary assay, the soils from 5 replicate subplots were pooled as one composite sample for each treatment, and only triplicate aliquots of each sample were subjected to elemental analyses. Total organic carbon (TOC) levels were measured using a TOC analyzer (TOC-L, Shimadzu, Japan). Total nitrogen (TN) levels were analyzed using a fully automatic azotometer (Kjeltec, 2300, Foss, Sweden).

### Statistical Analyses

Soil chemical characteristics, the relative abundances of bacteria and fungi, and α-diversity indices between no wheat straw return soils and wheat straw return soils were compared using Wilcoxon rank-sum test (*P* < 0.05). The relative abundances of nitrogen-cycling bacteria and plant pathogen-associated microbes were compared also using the Wilcoxon rank-sum test (*P* < 0.05). Spearman’s rank-correlation coefficient was used to evaluate the relationships between α-diversity indices and soil characteristics. All statistical analyses were performed using SPSS v. 20.0 (SPSS Inc.). The compositions of the microbial communities in N and R soils were compared using the Wilcoxon rank-sum test in the R package Stats.

## Results

### Effects of Wheat Straw Return on α-Diversity of Soil Microbial Communities

**FIGURE 1:**
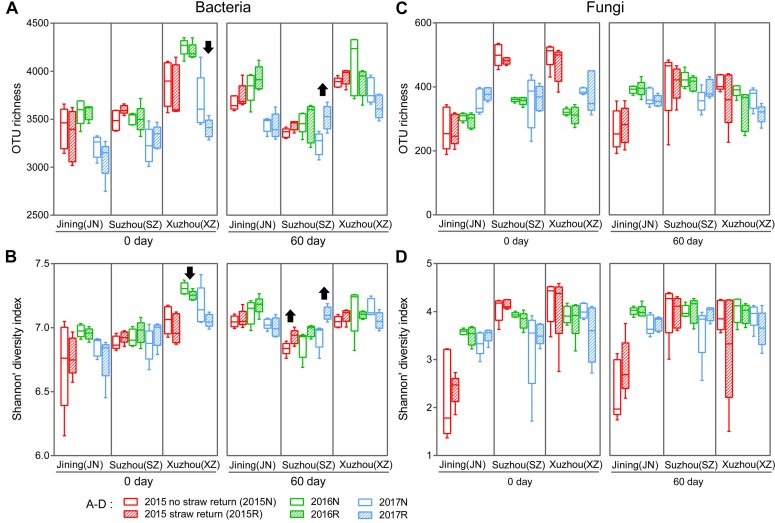
Richness (Sobs index) and α-diversity (Shannon’s diversity index) of the bacterial **(A**,**B)** and fungal **(C**,**D)** communities in N and R soils. Horizontal bars in boxes are medians; upper and lower box edges are the 75th and 25th quartiles, respectively; and whiskers are 1.5-fold the interquartile range. Wilcoxon rank-sum test results were displayed above the top whiskers; Significant differences between treatments and/or between years within a site and a period can be distinguished with different labels (*P* < 0.05, Wilcoxon rank-sum test).

We characterized the bacterial communities in the 180 bulk soil samples by sequencing the V4 region of the 16S rRNA gene. In all, 6,610,424 high-quality sequence reads were obtained with a median of 36,272 (range 30,312–44,663) per sample. The high-quality reads were clustered into 649,012 microbial OTUs based on a >97% sequence identity threshold. Measures of within-sample diversity (α-diversity) based on rarefied OTU tables (30,312 sequences per sample) revealed that the 60 days soils samples exhibited stronger responses to straw return in bacterial communities diversity from 2015 to 2017 (Figures 1A,B), although the responses differed among the three sites, and was rarely statistically significant (Supplementary Figure S2). Significantly higher bacterial diversity was observed in R soil than in N soil in Suzhou (*P* < 0.05, Wilcoxon rank-sum test; the first and third years of the 60 days periods). By contrast, R soil in Xuzhou contained lower bacterial diversity than N soil (*P* < 0.05, Wilcoxon rank-sum test; the second years of the 0 day period). The α-diversity in Jining did not differ significantly between the N and R treatments; however, the 0 d period had a lower bacterial α-diversity than the 60 days period (*P* < 0.05, Wilcoxon rank-sum test) in Jining (Figures 1A,B).

We also evaluated the fungal communities in the 180 bulk soil samples by sequencing of the fungal ITS1 region. In all, 6,920,939 high-quality sequences were obtained with a median read count per sample of 36,272 (range 14,959–44,984), and 66,471 microbial OTUs were obtained. Measures of α-diversity revealed a fungal community-diversity gradient between N and R, calculated based on rarefied OTU tables (14,959 sequences per sample). The fungal α-diversity values did not differ significantly between N and R treatments in the three fields (*P* > 0.05, Wilcoxon rank-sum test; Figures 1C,D).

### Effects of Wheat Straw Return on β-Diversity of Soil Microbial Communities

**FIGURE 2:**
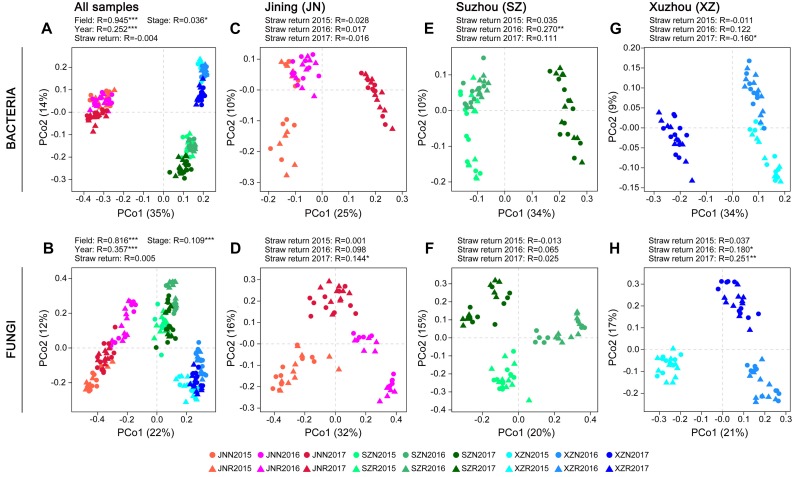
Principal coordinates analysis on bacterial and fungal community differences (Bray–Curtis dissimilarities) in the groups of samples. PCoAs showing the influence of field location and year on bacterial **(A)** and fungal **(B)** community composition. PCoAs of bacterial and fungal community composition in soils from different field locations **(C–H)**. ANOSIM was applied to test for differences in community composition due to straw return and field location. *R* values are shown with ^*^P < 0.05, ^∗∗^*P* < 0.01, and ^∗∗∗^*P* < 0.001.

Differences in the composition of the microbial communities among samples and between groups of samples (β-diversity) were analyzed based on principal coordinate analysis (PCoA) and ANOSIM. Because of the strongest differences due to field location (Figures 2A,B), the samples from each site were analyzed separately to assess the impact of straw return in more detail. The response of bacterial and fungal community composition to wheat straw return was stronger in 60 days than in 0 day. Higher correlation to the factor of straw return were observed in fungal communities than in bacterial communities, especially in JN and XZ soils, and the correlation values were both increased year by year. Although the SZ soils exhibited stronger responses to straw return in both bacterial and fungal communities with a peak value at the second year, they exhibited decreased correlation values in bacterial communities year by year in 60 days (Figures 2C–H and Supplementary Table S1).

### Differential Abundance of Bacterial and Fungal Taxa Under Wheat Straw Return

**FIGURE 3:**
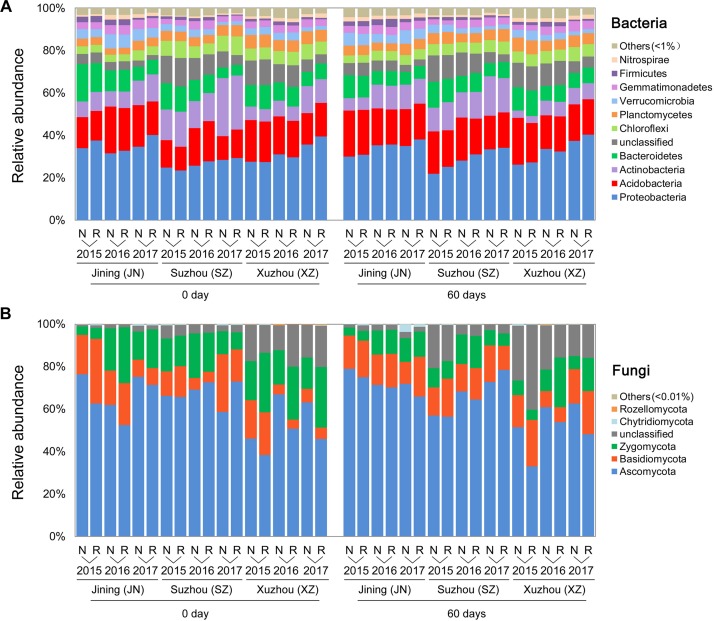
Effects of wheat straw return on the composition of the soil microbial community. Relative abundances of the most abundant bacterial **(A)** and fungal **(B)** phyla in each stage, site, year, and straw treatment.

In a phylum-level analysis, bacterial OTUs were classified into 45 phyla, predominantly Proteobacteria (31.6%), Acidobacteria (17.3%), Actinobacteria (10.9%), Bacteroidetes (9.0%), Chloroflexi (5.4%), and Planctomycetes (4.8%) (Figure 3A); these accounted for 86.5% of the bacterial sequences. Acidobacteria were more abundant in N soils, and Proteobacteria were more common in R soils. Fungal OTUs were predominantly associated with the phyla Ascomycota, Basidiomycota, and Zygomycota, which accounted for 90.3% of the total fungal sequences (Figure 3B). The relative abundance of Zygomycota was higher in R than in N soils, whereas that of Ascomycota showed the opposite trend (*P* < 0.05, Wilcoxon test).

**FIGURE 4:**
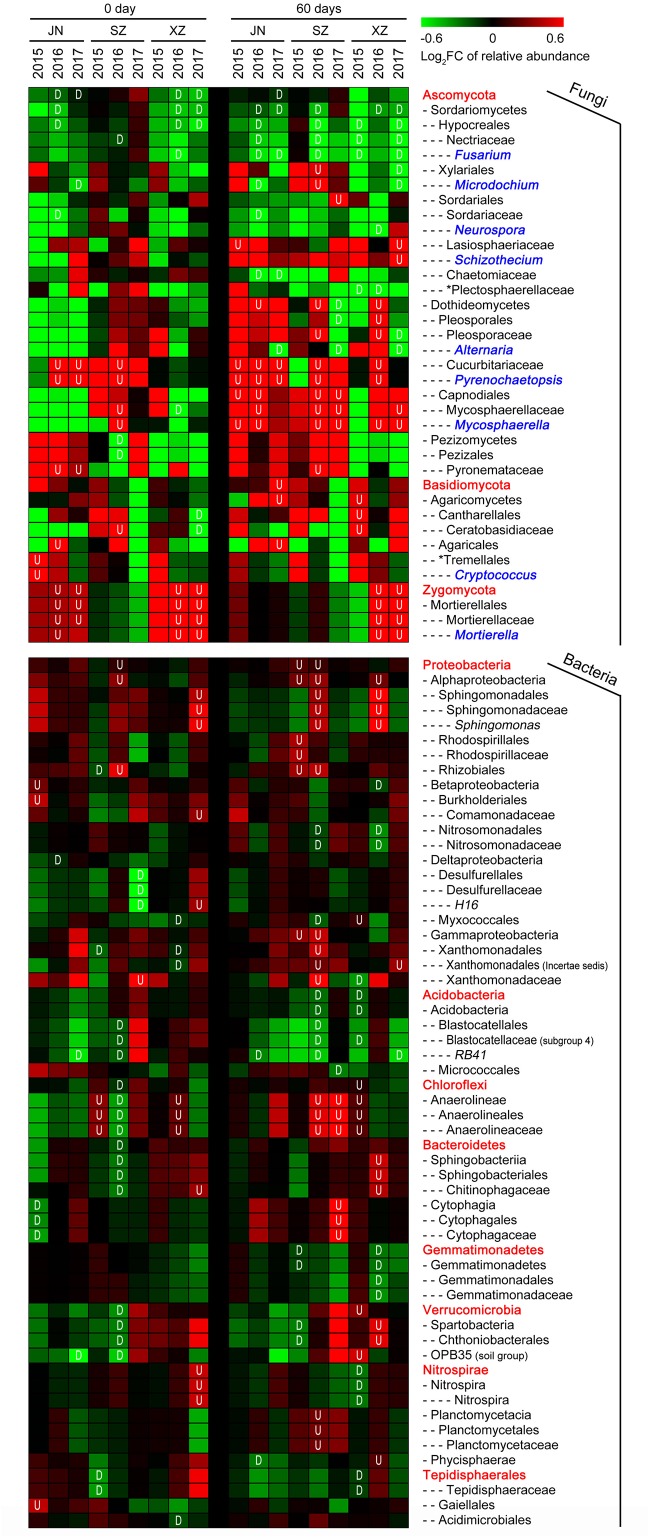
Straw return-responsive taxa (*P* < 0.05) of fungi and bacteria in each stage, site and year. The color of the cell indicates the log_2_ fold change in relative abundance with respect to the control treatment: an increase tends toward red, while a decrease tends toward green. The short dashes in the heatmap indicate the class (-), order (–), family (—), and genus (—-), the red fonts without short dash indicate the phylum level. Fungal genera marked in blue fonts. The plot displays all taxa detected as significantly affected by straw treatment in at least one site or year. “U” represents the relative abundance significantly increased in R soils, and “D” represents the relative abundance significantly decreased in R soils.

We further assessed the impact of straw return on the abundances of individual OTUs (relative abundance > 1%). For fungi, straw return led to an enrichment of OTUs belonging to classes Mortierellales (phylum Zygomycota) and Dothideomycetes (phylum Ascomycota) (Figure 4). The phylum with the most straw return-depleted OTUs across all site soils was Ascomycota. The majority of these OTUs were classified as Sordariomycetes, mostly represented by the family Nectriaceae and genus *Fusarium* (Figure 4). Thus, the straw return-responsive OTUs generally followed a consistent trend within certain phyla, classes, orders, families, and genera. For bacteria, among straw return-enriched OTUs, Proteobacteria was the most highly represented phylum in R soil communities (Figure 4). The relative abundances of several OTUs classified as Chloroflexi, mainly from the class Anaerolineae, increased under straw return. Within Proteobacteria, most straw return enriched OTUs were classified as Alphaproteobacteria and Gammaproteobacteria. In particular, Alphaproteobacteria was mainly represented by the orders Sphingomonadales and Rhizobiales (mostly in SZ and XZ soils). The phylum with the most straw return-depleted OTUs across all sites was Acidobacteria (Figure 4). The majority of these OTUs were classified as Acidobacteria, with Acidobacteria and Blastocatellales being the orders most broadly affected. OTUs from the class Gemmatimonadetes (phylum Gemmatimonadetes) were also depleted by straw return in SZ and XZ soils.

**FIGURE 5:**
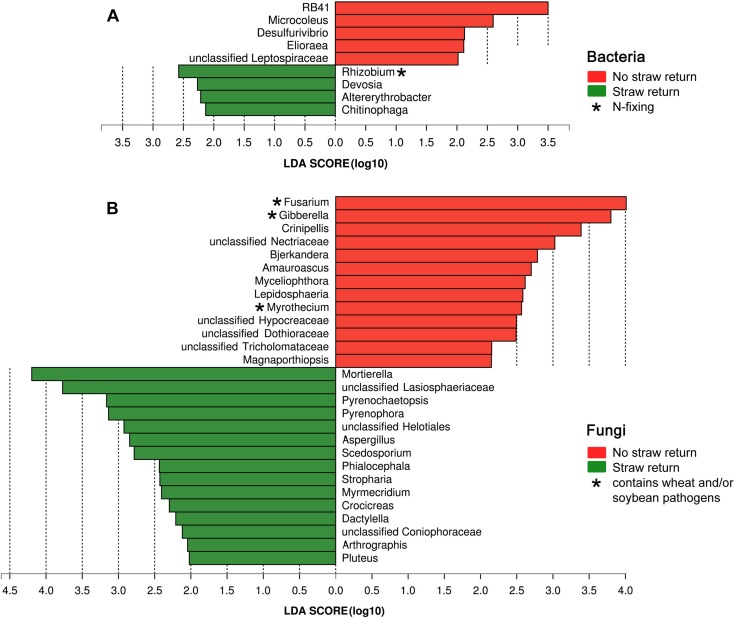
Histogram of the LDA scores computed for differentially abundant bacterial **(A)** and fungal **(B)** genera between the N and R soils.

Based on the LDA effect size (LEfSe) method, we identified several bacterial, and fungal genera which were significantly associated with wheat straw return. The bacterial genera *Rhizobium*, *Devosia*, *Altererythrobacter*, and *Chitinophaga* were more abundant in R soils, and *RB41*, *Microcoleus*, *Desulfurivibrio*, and *Elioraea* were more common in N soils (Figure 5A). The fungal genera *Mortierella*, *Pyrenochaetopsis*, *Pyrenophora*, *Aspergillus*, *Scedosporium*, *Phialocephala*, *Stropharia*, *Myrmecridium*, *Crocicreas*, *Dactylella*, *Arthrographis*, and *Pluteus* were more abundant in R soils, and *Fusarium*, *Crinipellis*, *Bjerkandera*, *Amauroascus*, *Myceliophthora*, *Lepidosphaeria*, *Myrothecium*, and *Magnaporthiopsis* were more common in N soils (Figure 5B). Interestingly, the bacterial genus *Rhizobium* was related to nitrogen cycling (Figure 5A), and the fungal genera *Fusarium* and *Myrothecium* contain pathogens of soybean or wheat (Figure 5B).

### Microbes Involved in the Nitrogen Cycling

**FIGURE 6:**
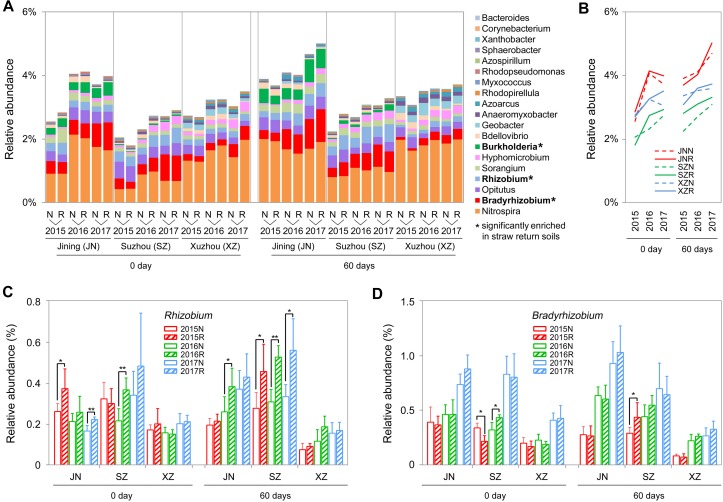
OTUs involved in the nitrogen cycle in N and R soils from different sites. **(A)** Detailed relative abundances of 19 nitrogen cycle-related genera in indicated samples. Asterisk indicates a significant difference with *P* < 0.05 (Wilcoxon rank-sum test). **(B)** Accumulated relative abundance of 19 nitrogen cycle-related genera in N and R soils during the three-season experiment. **(C**,**D)** Relative abundance of the genera *Rhizobium* and *Bradyrhizobium*, respectively. Asterisk indicates a significant difference with *P* < 0.05 (Wilcoxon rank-sum test).

We further identified 19 genera associated with nitrogen cycling from the sequencing results, according to the description of previous reports (Nelson et al., 2016 and Supplementary Table S2). In most of the cases (72%), the total abundance of these nitrogen cycle-related bacteria was higher in R soils than in N soils, and among the three sites, the overall increasing rates in R soils were higher than in N soils during the 3 years, particular at 60 days (Figures 6A,B). Among these bacteria, the total abundance of nitrogen-fixing bacteria (*Bradyrhizobium*, *Rhizobium*, and *Burkholderia*) and denitrifying bacteria (*Hyphomicrobium*) was significantly increased by straw return. For example, *Rhizobium* was significantly enriched in R soils of Jining and Suzhou, and *Bradyrhizobium* was significantly enriched in R soils of Suzhou (Figures 6A,C,D).

### Microbes Related to Wheat and/or Soybean Pathogens

**FIGURE 7:**
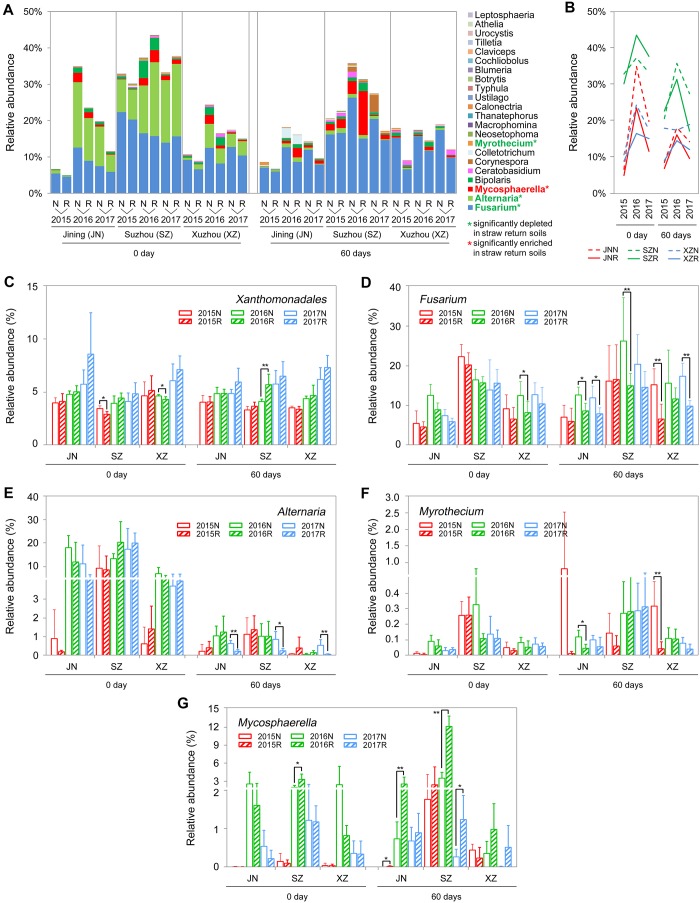
OTUs involved in wheat or soybean pathogen-associated genera in N and R soils from different sites. **(A)** Detailed relative abundance of 23 fungal genera in indicated sample. Asterisk indicates a significant difference with *P* < 0.05 (Wilcoxon rank-sum test). **(B)** Accumulated relative abundance of 23 fungal genera in N and R soils during the three-season experiment. **(C–G)** Relative abundance of the bacteria order Xanthomonadales and the fungal genera *Fusarium*, *Alternaria*, *Myrothecium*, and *Mycosphaerella*, respectively. Asterisk indicates a significant difference with *P* < 0.05 (Wilcoxon rank-sum test).

Comparing to the R soils, the N soils exhibited higher abundances of fungal genera that contain wheat and/or soybean pathogens, such as *Fusarium*, *Alternaria*, and *Myrothecium* (Bai et al., 2015; Zeng et al., 2016; Dewangan et al., 2017). In our amplicon sequencing annotation results, we identified 23 fungal genera and one bacterial genus containing plant pathogens. The 23 fungal genera were of the phyla *Ascomycota* and Basidiomycota. The relative abundances of these genera were low (<1%), with the exceptions of *Fusarium*, *Alternaria*, and *Mycosphaerella* (Figure 7A). Surprisingly, straw return decreased the total abundance of pathogenic fungi (Figures 7A,B) but increased that of the pathogenic bacterium *Xanthomonadales* (Figure 7C). In detail, the relative abundances of the pathogenic fungal genera *Fusarium*, *Alternaria*, and *Myrothecium* were significantly lower in R soils, while that of *Mycosphaerella*, which was significantly higher (*P* < 0.05, Wilcoxon rank-sum test) (Figures 7A,D–G).

### Effects of Soil Total Organic Carbon and Total Nitrogen on Microbial Communities

**FIGURE 8:**
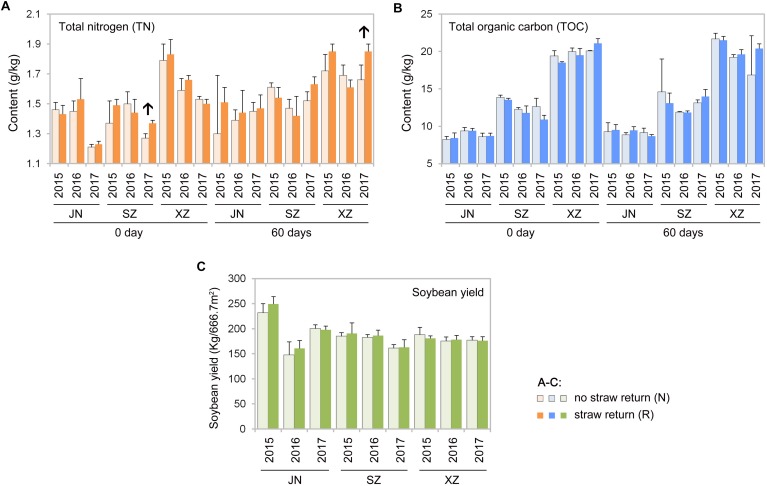
Total nitrogen **(A)** and total organic carbon **(B)** contents in the indicated soil samples, and soybean yields between no straw return and straw return fields **(C)**. Significant differences between the treatments are indicated by arrows (*P* < 0.05, Wilcoxon rank-sum test).

Mantel tests revealed that contents of TOC and TN in soil were significantly correlated with the composition of the soil bacterial and fungal communities (*R* = 0.61, *P* < 0.01 for bacteria and *R* = 0.57, *P* < 0.01 for fungi). According to Spearman’s rank-correlation coefficients, the bacterial richness and diversity, and the fungal diversity but not richness, were significantly correlated with soil TOC and TN contents (*P* < 0.01). TOC and TN contents were slightly different between R than N soils; however, some results indicated that there might be tendency that TN content was increased after wheat straw return. For example, TN content was significantly higher in R than N soils of SZ (0 day) and XZ (60 days) collected in 2017 (*P* < 0.05, Wilcoxon rank-sum test; Figures 8A,B).

## Discussion

Despite its widespread applications to improve soil fertility and crop yields, the effects of wheat straw return on soil microbial communities in a wheat–soybean rotation system was unclear. Our results of investigation in the Huang-Huai region showed that wheat straw return altered the taxonomic and functional traits of soil microbial communities, including increases in the relative abundances of nitrogen cycle-related bacterial taxa and decreases in those of some pathogenic fungal taxa. These shifts are likely to impact belowground ecosystems.

To evaluate the consistent effects of straw return on soil microbial communities, we collected samples from three different fields. Comparing to the effects of straw return, the effects of field location was stronger. The bacterial and fungal α-diversity differed among the three sites, and the primary source of β-diversity was the field location (Figures 1, 2). This is expected and probably due to the differences of soil type and environmental factors (Brockett et al., 2012; Peiffer et al., 2013; Edwards et al., 2015; Almario et al., 2017). In addition, the response of the α-diversity of the soil bacterial community to wheat straw return differed among the three sites (Supplementary Figure S1), probably due to the influence of soil texture and agronomic management history (Cookson et al., 2008; Bach et al., 2010; Xiong et al., 2015; Zhang et al., 2017). Furthermore, planting year and sampling period affected the soil bacterial and fungal communities (Figures 1, 2). These results are in line with prior reports (Sugiyama et al., 2014; Zeng et al., 2014; Bai et al., 2015; Shen et al., 2015; Dang et al., 2017).

Based on the samples collected from three different fields across three seasons, we found that wheat straw return affected the relative abundance and diversity of the overall soil bacterial community (Figures 1A,B). Although there was no significant overall effect on fungal community (Figures 1C,D), stronger responses of fungal communities to wheat straw return were observed, especially in the second, and third years (Figures 2C–H and Supplementary Table S1). This may because that the life generation cycle of bacteria is more rapid than fungi, and bacteria dominate in the initial phases of crop straw decomposition and fungi dominate the latter stage (Poll et al., 2008; Marschner et al., 2011; Chen Z. et al., 2017). Furthermore, soil bacteria dominated the decomposition of crop residue in agricultural soils, and fungi were the main decomposers in prairie soils (Allison et al., 2005). The short-term responses of soil microbial communities to wheat straw return were relatively weak compared to the other factors, they became evident after excluding the variation caused by field location and year. For example, wheat straw return enhanced the relative abundance of the bacterial phylum Proteobacteria and the fungal phylum Zygomycota, but reduced that of the bacterial phylum Acidobacteria and the fungal phylum Ascomycota (Figures 3A,B). The Proteobacteria to Acidobacteria ratio has been suggested to be an indicator of the trophic level of soils (Smit et al., 2001; Castro et al., 2010; Gottel et al., 2011; Beckers et al., 2017), as Proteobacteria are favored in nutrient-rich soils and Acidobacteria in nutrient-poor soils. Alphaproteobacteria genera such as *Rhizobium*, *Devosia*, and *Altererythrobacter* were more abundant in R soils, and *RB41* (*Acidobacteria*) were more common in N soils (Figure 5A). Ascomycota was more abundant in N soils, whereas Zygomycota was more common in R soils. Ascomycota includes various soil-borne plant-pathogenic fungi as well as non-pathogenic genera, and its relative abundance is higher in areas of low productivity (Chang et al., 2017). Among them, the relative abundances of *Fusarium* were significantly lower in R soils. Zygomycota is reportedly rare in agricultural soils (Huang et al., 2015; Wang et al., 2015). However, dominant Zygomycota species such as *Mortierella* may play a role in disease suppression (Tagawa et al., 2010; Wani et al., 2017; Xiong et al., 2017). In our results, *Mortierella* was the most abundant genus in R soils. These results indicated that wheat straw return could be beneficial to soil health.

Wheat straw return enriched bacteria genera related to the soil nitrogen cycle, including *Nitrospira*, *Bradyrhizobium*, *Rhizobium*, and *Hyphomicrobium* (Figure 6A). *Nitrospira* were thought to be capable only of nitrite oxidation but recent evidence suggests that they may oxidize ammonia (Daims et al., 2015; Longa et al., 2017; Kuypers et al., 2018). *Bradyrhizobium* and *Rhizobium* are related to nodulation, and their symbiosis with legumes enhances nitrogen fixation and increases crop productivity (Sugiyama et al., 2014; Chang et al., 2017; Kuypers et al., 2018). *Hyphomicrobium* has denitrifying activity and is widely used in wastewater treatment (Rissanen et al., 2016; Sun et al., 2017; Yasuda et al., 2017). The relative abundances of *Bradyrhizobium* and *Hyphomicrobium* have been reportedly to be higher in a straw-returned than control (no straw return) soil in the North China Plain (Chen Y. et al., 2017). In additional to microbial community traits, soil characteristics may also be affected by wheat straw return. In this study, TN contents were moderately higher in R than N soils (Figure 8A). This is in line with a previous report that wheat straw return significantly increased the TN content compared to the control treatment (Chen Y. et al., 2017). Although we found that wheat straw return did not significantly influence the soil TOC content (Figure 8B), we speculated that impacts of straw return on soil C and N contents are strongly dependent on initial soil C and N, the rates and duration of straw return, and local soil and climate conditions.

Wheat straw return significantly reduced the abundances of plant pathogen associated fungal genera such as *Fusarium*, *Alternaria*, and *Myrothecium* (Figures 7A,D,E,F). Some *Fusarium* species can cause *Fusarium* root rot of soybean and *Fusarium* head blight of wheat both diseases markedly decrease crop productivity (Bai et al., 2015; Cainong et al., 2015; Lenc, 2015; Lu et al., 2015). Similarly, some *Alternaria* species can cause leaf-spot disease of soybean, sunflower, *Withania somnifera*, and *Pelargonium* (Furukawa and Kishi, 2001; Oliveira et al., 2004; Pati et al., 2008; Kumar and Kumar, 2017). Plant-pathogenic *Myrothecium* species cause foliar spots in soybean, cotton, *Anthurium*, *Nicandra physaloides*, tomato, and cucumber (Quezado Duval et al., 2010; Kwon et al., 2014; Dewangan et al., 2017). However, the relative abundances of other microbial taxa that include some plant pathogens, such as *Mycosphaerella*, were enhanced in R soils (Figure 7G). *Mycosphaerella* species can cause leaf-spot disease in economically important crops including wheat, soybean, banana, citrus, and eucalyptus (Stukenbrock et al., 2011; Ohm et al., 2012; Zeng et al., 2017). In addition, the relative abundance of the bacterial order Xanthomonadales was increased in R soils (Figure 7C). Xanthomonadales species cause major losses of tomato, cabbage, pepper, banana, citrus, rice, grape, peach, plum, almond, coffee, and maple crops (Ryan et al., 2011; Naushad and Gupta, 2013; Garita-Cambronero et al., 2018).

Straw return is often considered to increase pathogen loads and promote diseases of crops (Li et al., 2012; Qiao et al., 2013). Yet, there are other examples where long-term straw return suppressed some plant diseases and promote healthy plant growth (Osunlaja, 1990; Bailey and Lazarovits, 2003). Tillage and straw incorporation into soil may reduce pathogen inocula, while facilitating nutrient cycling, probably because no-tillage, and straw-returning practices improving soil nutrition condition for soil microbial communities (Guo et al., 2016). In our 3-yr study, as an indication of plant healthy, crop yield in R soils did not differ significantly than that in N soils every year (Figure 8C). The lower pathogenic fungal diversity in our straw return soil may be to the result of the lower returning amount (ca.5000 kg.hm^–2^) of wheat straw before soybean production. Some previous studies observed that the occurrence of soil-borne diseases was significantly different under different straw amendment rates. For example, the indexes of wheat soil-borne diseases were reduced significantly at the maize straw amendment rates of 7500 and 3750 kg.hm^–2^), but increased dramatically when the amendment rate increased to 15000 kg/hm^2^ (Zhen et al., 2009). In our study, although the abundance of pathogenic fungi in R soil is lower than that in N, abundances of many fungal genera in R is still very high, and straw return could increase soil moisture, which may further promote the occurrence of diseases. Future studies using a series of root-associated compartments experiments would be helpful for disentangling the relationships between soil microbiota and straw return.

## Conclusion

Our results from a short-term, multisite experiment showed that wheat straw return could alter the taxonomic, and functional traits of the soil bacterial and fungal communities. The bacterial phylum Proteobacteria and the fungal phylum Zygomycota were enriched in R soils, but the bacterial phylum Acidobacteria, and the fungal phylum Ascomycota were depleted. Wheat straw return enriched the population of bacteria related to the soil nitrogen cycle, such as *Bradyrhizobium* and *Rhizobium*. Wheat straw return significantly depleted plant pathogen associated fungal taxa, such as *Fusarium* and *Alternaria*. R soils had moderately higher TN contents than N soils. Long-term experiments are needed to examine whether our observed changes in the microbial community composition induced by wheat straw return persist over time.

## Data Availability

The datasets generated for this study can be found in NCBI, BioProject: PRJNA524011.

## Author Contributions

HY performed the experiments, analyzed the data, contributed reagents, materials, and analysis tools, prepared the figures and/or tables, and drafted the manuscript and approved its final version. JM performed the experiments, contributed reagents, materials, and analysis tools, and approved the final version of the manuscript. ZR and DZ contributed reagents, materials, and analysis tools, and approved the final version of the manuscript. YW conceived and designed the experiments, and reviewed the drafted manuscript and approved its final version. SH reviewed the drafted manuscript and approved its final version. WY conceived and designed the experiments, prepared the figures and/or tables, and reviewed the drafted manuscript and approved its final version. XZ conceived and designed the experiments and approved the final version of the manuscript.

## Conflict of Interest Statement

The authors declare that the research was conducted in the absence of any commercial or financial relationships that could be construed as a potential conflict of interest.

## Footnotes

1 https://www.mothur.org

2 https://www.r-project.org

3 http://qiime.org

4 http://huttenhower.sph.harvard.edu/galaxy

## Supplementary Material

FIGURE S1 Field experiment design. **(A)** The geographic coordinates of the three selected sites in the Huang-Huai region of China. **(B)** Schematic representation of field experiment layout. Each site had two treatments. Each treatment contained five replicated subplots.

FIGURE S2 Bacterial and fungal alpha diversity estimated by Shannon’s diversity index. Significant differences between the treatments are distinguished by different letters (*P* < 0.05, Wilcoxon rank-sum test).

TABLE S1 Influence of straw return on microbial community composition according to *R*-values derived from an ANOSIM. *R* values are shown with ^*^*P* < 0.05, ^∗∗^*P* < 0.01, and ^∗∗∗^*P* < 0.001.

TABLE S2 Annotation of the 19 nitrogen cycling related bacterial genera according to the description of previous reports.
