# Supplementary figures and images for: Wheat Straw Return Influences Nitrogen-Cycling and Pathogen Associated Soil Microbiota in a Wheat–Soybean Rotation System
Source: Front Microbiol. 2019 Aug 8;10:1811. doi: 10.3389/fmicb.2019.01811 (PMC6694757; doi:10.3389/fmicb.2019.01811)

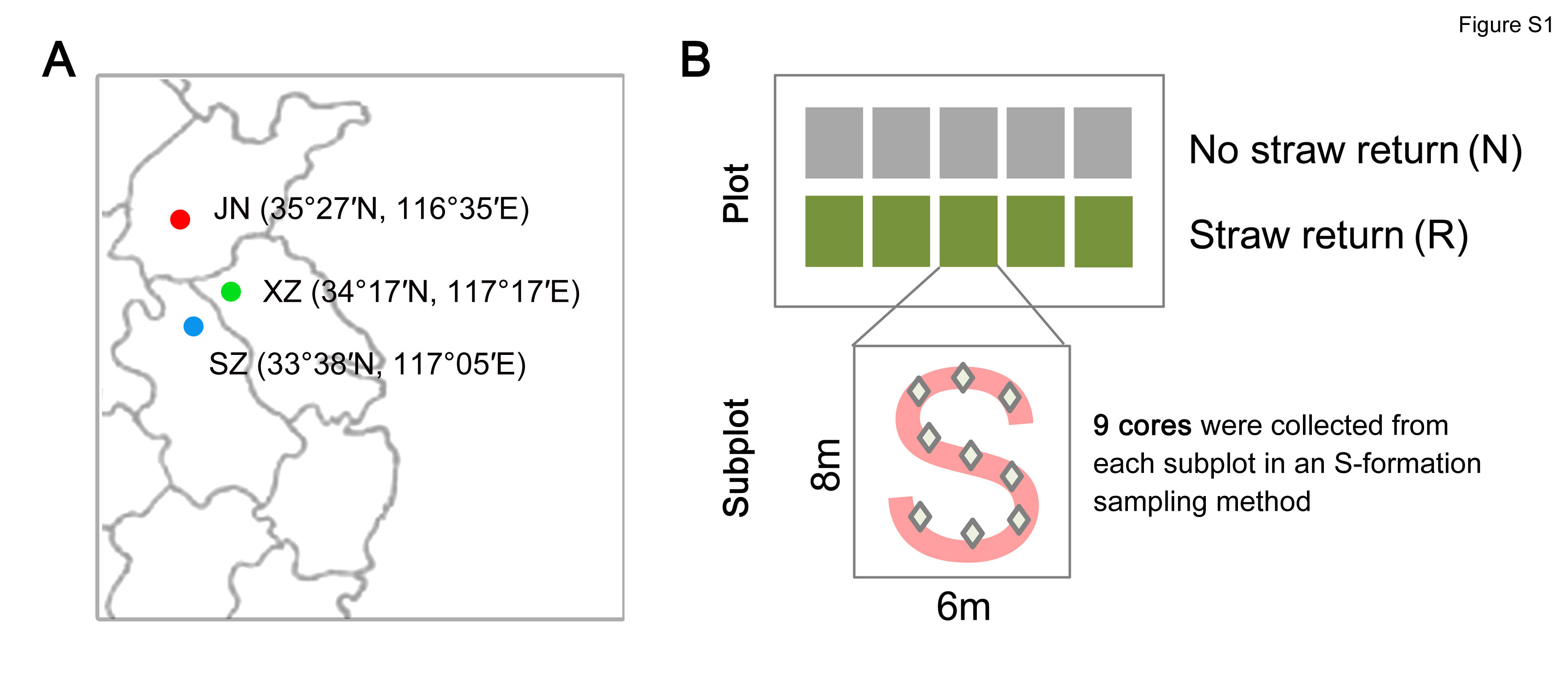

Supplement: FIGURE S1 — Field experiment design. (A) The geographic coordinates of the three selected sites in the Huang-Huai region of China. (B) Schematic representation of field experiment layout. Each site had two treatments. Each treatment contained five replicated subplots. [file Image_1.JPEG]

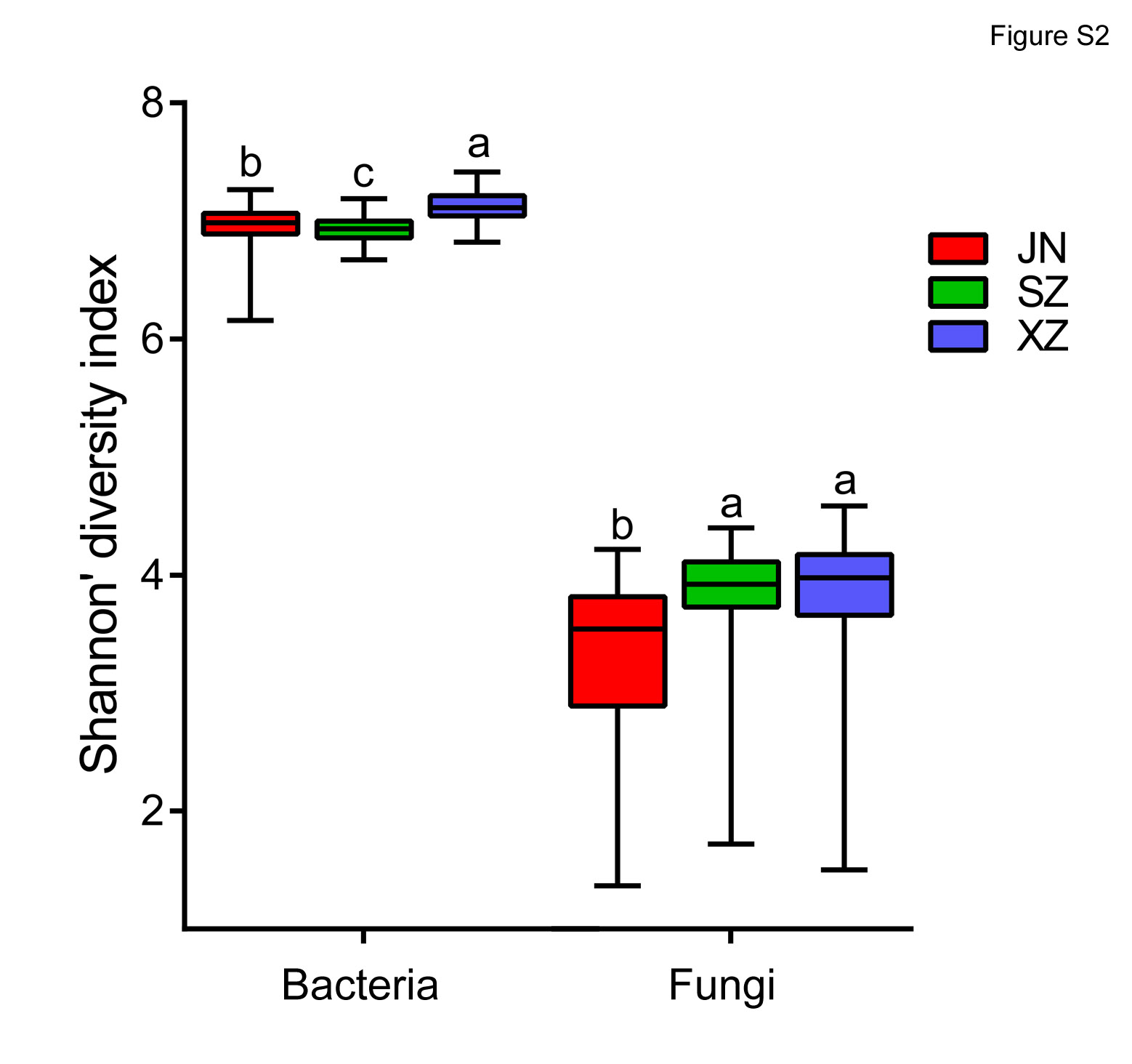

Supplement: FIGURE S2 — Bacterial and fungal alpha diversity estimated by Shannon’s diversity index. Significant differences between the treatments are distinguished by different letters (P < 0.05, Wilcoxon rank-sum test). [file Image_2.JPEG]
