# Supplementary material for: Wheat Straw Return Influences Nitrogen-Cycling and Pathogen Associated Soil Microbiota in a Wheat–Soybean Rotation System
Source: Front Microbiol. 2019 Aug 8;10:1811. doi: 10.3389/fmicb.2019.01811 (PMC6694757; doi:10.3389/fmicb.2019.01811)
Supplement: TABLE S1 — Influence of straw return on microbial community composition according to R-values derived from an ANOSIM. R values are shown with *P < 0.05, ∗∗P < 0.01, and ∗∗∗P < 0.001. [file Table_1.DOCX]

**Table S1.** Influence of straw return on microbial community composition according to R-values derived from an ANOSIM. R values are shown with ∗ for P < 0.05, ∗∗ for P < 0.01, and ∗∗∗ for P < 0.001.

| Year | Site | Bacteria | |  | Fungi | |
| --- | --- | --- | --- | --- | --- | --- |
|  |  | 0 d | 60 d |  | 0 d | 60 d |
| 2015 | JN | 0.076 | -0.044 |  | 0.004 | -0.092 |
|  | SZ | 0.230 | 0.210 |  | -0.132 | 0.052 |
|  | XZ | -0.060 | 0.594^**^ |  | -0.072 | 0.232 |
| 2016 | JN | -0.018 | 0.140 |  | 0.408^*^ | 0.364^**^ |
|  | SZ | 0.796^*^ | 0.576^**^ |  | 0.278 | 0.676^*^ |
|  | XZ | 0.146 | 0.340^*^ |  | 0.220 | 0.276^*^ |
| 2017 | JN | 0.040 | 0.098 |  | 0.056 | 0.366^*^ |
|  | SZ | 0.064 | 0.308^*^ |  | 0.156 | 0.396^**^ |
|  | XZ | 0.194 | 0.168 |  | 0.252 | 0.524^**^ |

**Table S2.** Annotation of the 19 nitrogen cycling related bacterial genera according to the description of previous reports

| Genera | References | Description |
| --- | --- | --- |
| *Bacteroides* | 1, 11 | dissimilatory nitrite reduction to ammonium |
| *Corynebacterium* | 1, 12 | dissimilatory nitrite reduction to ammonium;  nitrate–nitrite conversion |
| *Xanthobacter* | 1, 13, 14, 27 | nitrogen fixation;  catalysing the reduction of nitric oxide to nitrous oxide |
| *Sphaerobacter* | 1, 15 | dissimilatory nitrite reduction to ammonium;  nitrite-oxidizing bacterium |
| *Azospirillum* | 1, 16, 17 | nitrogen fixation |
| *Rhodopseudomonas* | 1, 18, 19, 20 | nitrogen fixation |
| *Myxococcus* | 1, 21, 26 | dissimilatory nitrite reduction to ammonium |
| *Rhodopirellula* | 1, 22 | dissimilatory nitrite reduction to ammonium |
| *Azoarcus* | 1,23, 24 | nitrogen fixation |
| *Anaeromyxobacter* | 1, 2 | dissimilatory nitrite reduction to ammonium |
| *Geobacter* | 1, 25 | dissimilatory nitrite reduction to ammonium;  fix N_2_ |
| *Bdellovibrio* | 1, 21, 26 | dissimilatory nitrite reduction to ammonium |
| *Burkholderia* | 1, 6, 7 | nitrogen fixation |
| *Hyphomicrobium* | 8, 9, 10 | denitrifying activity |
| *Sorangium* | 1, 27 | dissimilatory nitrite reduction to ammonium;  catalysing the reduction of nitric oxide to nitrous oxide |
| *Rhizobium* | 6, 7 | nitrogen fixation |
| *Opitutus* | 1, 28, 29 | dissimilatory nitrite reduction to ammonium;  denitrifiers |
| *Bradyrhizobium* | 1, 5, 6, 7 | nitrogen fixation |
| *Nitrospira* | 3, 4, 5 | complete nitrification |

**REFERENCES**

1. Nelson, M. B., Martiny, A. C., & Martiny, J. B. H. (2016). Global biogeography of microbial nitrogen-cycling traits in soil. Proceedings of the National Academy of Sciences, 113(29), 8033-8040.
2. Sanford RA, et al. (2012) Unexpected nondenitrifier nitrous oxide reductase gene diversity and abundance in soils. Proc Natl Acad Sci USA 109(48):19709–19714.
3. Daims, H., Lebedeva, E.V., Pjevac, P., Han, P., Herbold, C., Albertsen, M., et al. (2015). Complete nitrification by *Nitrospira* bacteria. Nature 528(7583), 504-509. doi: 10.1038/nature16461.
4. Coskun, D., Britto, D. T., Shi, W., & Kronzucker, H. J. (2017). How plant root exudates shape the nitrogen cycle. Trends in Plant Science, S1360138517300936.
5. Mmm, K., Marchant, H. K., & Kartal, B. (2018). The microbial nitrogen-cycling network. Nature Reviews Microbiology, 16(5).
6. Feng, M., Adams, J. M., Fan, K., et al. (2018). Long-term fertilization influences community assembly processes of soil diazotrophs. Soil Biology and Biochemistry, 126, 151-158.
7. Peix, A., Ramírez-Bahena, M. H., Velázquez, E., & Bedmar, E. J. (2015). Bacterial associations with legumes. Critical Reviews in Plant Sciences, 34(1-3), 17-42.
8. Rissanen, A.J., Ojala, A., Fred, T., Toivonen, J., and Tiirola, M. (2017). *Methylophilaceae* and *Hyphomicrobium* as target taxonomic groups in monitoring the function of methanol-fed denitrification biofilters in municipal wastewater treatment plants. J Ind Microbiol Biotechnol 44(1), 35-47. doi: 10.1007/s10295-016-1860-5.
9. Sun, H., Liu, F., Xu, S., Wu, S., Zhuang, G., Deng, Y., et al. (2017). *Myriophyllum aquaticum* Constructed Wetland Effectively Removes Nitrogen in Swine Wastewater. Front Microbiol 8, 1932. doi: 10.3389/fmicb.2017.01932.
10. Yasuda, T., Waki, M., Fukumoto, Y., Hanajima, D., Kuroda, K., Suzuki, K., et al. (2017). Community structure of denitrifying and total bacteria during nitrogen accumulation in an ammonia-loaded biofilter. J Appl Microbiol 123(6), 1498-1511. doi: 10.1111/jam.13603.
11. Sobko, T., Reinders, C., Jansson, EÅ, Norin, E., Midtvedt, T., & Lundberg, J. (2005). Gastrointestinal bacteria generate nitric oxide from nitrate and nitrite. Nitric Oxide, 13(4), 272-278.
12. Platzen, L., Koch-Koerfges, A., Weil, B., Brocker, M., & Bott, M. (2014). Role of flavohaemoprotein hmp and nitrate reductase narghji of corynebacterium glutamicum for coping with nitrite and nitrosative stress. FEMS Microbiology Letters,350(2), 239-248.
13. Arun, A. B., Schumann, P., Chu, H. I., Tan, C. C., Chen, W. M., & Lai, W. A., et al. (2008). *Pseudoxanthobacter soli* gen. nov. sp. nov. a nitrogen-fixing alphaproteobacterium isolated from soil. Int J Syst Evol Microbiol, 58(Pt 7), 1571-1575.
14. Liu, C., Sakimoto, K. K., Colón, Brendan C., Silver, P. A., & Nocera, D. G. (2017). Ambient nitrogen reduction cycle using a hybrid inorganic–biological system. Proceedings of the National Academy of Sciences, 201706371.
15. Sorokin, D. Y., Vejmelkova, D., Lucker, S., Streshinskaya, G. M., Rijpstra, W. I. C., & Sinninghe Damste, J. S., et al. (2014). *Nitrolancea hollandica* gen. nov. sp. nov. a chemolithoautotrophic nitrite-oxidizing bacterium isolated from a bioreactor belonging to the phylum chloroflexi. International Journal of Systematic and Evolutionary Microbiology, 64(Pt 6), 1859-1865.
16. Fukami, J., Cerezini, P., & Hungria, M. (2018). *Azospirillum*: benefits that go far beyond biological nitrogen fixation. AMB Express, 8(1), 73.
17. Pankievicz, V. C., do Amaral, F. P., Santos, K. F., Agtuca, B., Xu, Y., Schueller, M. J., ... & Stacey, G. (2015). Robust biological nitrogen fixation in a model grass–bacterial association. The Plant Journal, 81(6), 907-919.
18. Cantera, J. J. L., Kawasaki, H., & Seki, T. (2004). The nitrogen-fixing gene (*nifH*) of *Rhodopseudomonas palustris*: a case of lateral gene transfer? Microbiology, 150(7), 2237-2246.
19. Bentzon-Tilia, M., Severin, I., Hansen, L. H., & Riemann, L. (2015). Genomics and ecophysiology of heterotrophic nitrogen-fixing bacteria isolated from estuarine surface water. MBio, 6(4), e00929-15.
20. Arashida, H., Kugenuma, T., Watanabe, M., & Maeda, I. (2019). Nitrogen fixation in *Rhodopseudomonas palustris* co-cultured with *Bacillus subtilis* in the presence of air. Journal of bioscience and bioengineering, 127(5), 589-593.
21. Klotz, M. G., Schmid, M. C., Strous, M., Op Den Camp, H. J., Jetten, M. S., & Hooper, A. B. (2008). Evolution of an octahaem cytochrome c protein family that is key to aerobic and anaerobic ammonia oxidation by bacteria. Environmental microbiology, 10(11), 3150-3163.
22. Mohan, S. B., Schmid, M., Jetten, M., & Cole, J. (2004). Detection and widespread distribution of the *nrfA* gene encoding nitrite reduction to ammonia, a short circuit in the biological nitrogen cycle that competes with denitrification. FEMS Microbiology Ecology, 49(3), 433-443.
23. Chen, M. H., Sheu, S. Y., James, E. K., Young, C. C., & Chen, W. M. (2013). *Azoarcus olearius* sp. nov., a nitrogen-fixing bacterium isolated from oil-contaminated soil. International journal of systematic and evolutionary microbiology, 63(10), 3755-3761.
24. Sarkar, A., & Reinhold-Hurek, B. (2014). Transcriptional profiling of nitrogen fixation and the role of NifA in the diazotrophic endophyte *Azoarcus* sp. strain BH72. PLoS One, 9(2), e86527.
25. Bazylinski, D. A., Dean, A. J., Schüler, D., Phillips, E. J., & Lovley, D. R. (2000). N_2_-dependent growth and nitrogenase activity in the metal-metabolizing bacteria, *Geobacter* and *Magnetospirillum* species. Environmental microbiology, 2(3), 266-273.
26. Teske, A., Alm, E., Regan, J. M., Toze, S., Rittmann, B. E., & Stahl, D. A. (1994). Evolutionary relationships among ammonia-and nitrite-oxidizing bacteria. Journal of bacteriology, 176(21), 6623-6630.
27. Cameron, K. A., Hodson, A. J., & Osborn, A. M. (2012). Carbon and nitrogen biogeochemical cycling potentials of supraglacial cryoconite communities. Polar biology, 35(9), 1375-1393.
28. Peng, T., Feng, C., Hu, W., Chen, N., He, Q., Dong, S., ... & Li, M. (2018). Treatment of nitrate-contaminated groundwater by heterotrophic denitrification coupled with electro-autotrophic denitrifying packed bed reactor. Biochemical Engineering Journal, 134, 12-21.
29. Yoon, S., Cruz-García, C., Sanford, R., Ritalahti, K. M., & Löffler, F. E. (2015). Denitrification versus respiratory ammonification: environmental controls of two competing dissimilatory NO_3_^−^/NO_2_^−^ reduction pathways in *Shewanella loihica* strain PV-4. The ISME journal, 9(5), 1093.
